# Determining the factors associated with suicidal thoughts in outpatients referred to a specialized psychiatric facility

**DOI:** 10.1002/pcn5.70096

**Published:** 2025-04-25

**Authors:** Alireza Haji Seyed Javadi, Adeleh Askari Diarjani, Ali Akbar Shafikhani

**Affiliations:** ^1^ Clinical Research Development Unit Qazvin University of Medical Sciences 22 Bahman Hospital Qazvin Iran; ^2^ Department of Occupational Health Engineering Qazvin University of Medical Sciences Qazvin Iran

**Keywords:** mental disorders, psychopathology, socioeconomic factors, suicidal ideation

## Abstract

**Aim:**

Identifying the underlying factors that trigger suicidal ideation and understanding their interactive effects is essential for predicting suicidal thoughts. This study seeks to explore the psychopathological, socioeconomic, and demographic determinants of suicidal ideation in outpatients referred to a specialized psychiatric clinic.

**Methods:**

A cross‐sectional study was conducted at the psychiatric clinic of the 22nd Bahman Hospital in Qazvin, Iran, from 2020 to 2021. The study comprised 288 participants (78 with suicidal ideation and 210 without). All participants completed the Symptom Checklist‐90‐Revised (SCL‐90‐R) and Kuppuswamy's Socioeconomic Status Scale. Demographic and clinical data were collected and analyzed using appropriate statistical methods.

**Results:**

Individuals with suicidal ideation demonstrated significantly higher SCL‐90‐R scores compared to those without (150.84 ± 37.87 vs. 119.13 ± 33.81, respectively, *p* < 0.001). Among the SCL‐90‐R subscales, Obsessive‐Compulsive Disorder, Psychoticism, Depression, and Somatization exhibited the strongest correlations with suicidal ideation (*p* < 0.001). Significant risk factors for suicidal ideation included elevated SCL‐90‐R scores (odds ratio [OR] = 1.04, 95% confidence interval [CI]: 1.02–1.05), marital status (being married vs single) (OR = 0.13, 95% CI: 0.05–0.29), education level below diploma (OR = 2.95, 95% CI: 1.17–7.57), low socioeconomic status (OR = 5.80, 95% CI: 1.68–20.44), presence of personality disorder (OR = 3.86, 95% CI: 1.03–14.63), and major depression (OR = 6.40, 95% CI: 1.89–22.42) (*p* < 0.05).

**Conclusion:**

The results indicate that psychopathological symptoms, educational attainment, marital status, and socioeconomic challenges may contribute significantly to the development of suicidal ideation. It is recommended that specialized care clinics prioritize the assessment of suicidal thoughts to facilitate the implementation of appropriate preventive measures.

## INTRODUCTION

According to the World Health Organization report, more than 700,000 subjects die by suicide annually.^1^ Suicide is encountered in all age spectrums and was the fourth leading cause of death among youth aged 15–29 years in 2019.^2^, ^3^ One of the main challenges to psychological health in Iran is a rise in the suicide rate in some provinces.^4^ In Iran, the suicide rate has been reported to be eight per 100,000 subjects, with an average age of 32 years among victims.^5^, ^6^ Around 42% of Iranians committing suicide were reported to have a history of psychological disorders, and 40% declared the desire to commit suicide within the past year.^7^, ^8^

Suicide is a complicated phenomenon with multifactorial etiology. Suicide can be associated with psychological disorders, such as depression, bipolar disorder, schizophrenia, socioeconomic status, and demographic features, driving subjects toward suicide.^9^, ^10^ Stressful events, such as poor economic status, educational problems, and relationship problems, such as divorce, may predispose subjects to suicide.^11^

Suicide inflicts an enormous economic burden and shrinks human resources, causing social and economic losses for the individual, family, and society.^12^ Unlike committing suicide, suicidal thoughts and related factors have been less studied.^13^ Suicidal thoughts are an essential indicator of suicide. In 60% of cases, the transition from suicidal ideation to suicide attempt occurs within 1 year.^14^, ^15^ Because of limited access to the data related to the factors affecting suicidal thoughts, most available studies in the field have investigated the relevant determinants at the macro level, including psychological and sociological studies.^15^ Regarding the different political, social, economic, and cultural atmospheres in various countries, the triggers of suicidal ideation and their impacts vary among societies.^16^ Therefore, it is essential to recognize these triggers and their combinational effects to predict suicidal thoughts.

In Iran, suicidal thoughts have been studied mostly among students and soldiers.^17^ In a study of 470 students in Isfahan, 7.6% of them had suicidal thoughts.^17^ In another study on 1329 soldiers, 7% of the participants had suicidal thoughts.^14^ However, no study has been conducted on suicidal thoughts and their predictors in outpatients referred to psychiatric clinics. Since underlying psychological disorders are an important variable in suicide, it is essential to identify the factors affecting suicidal thoughts in these patients. Therefore, this study was conducted to identify the psychopathological, socioeconomic, and demographic predictors of suicidal thoughts in outpatients referred to a specialized psychiatric clinic. Recognizing the risk factors predicting suicidal thoughts can help develop a suitable and cost‐effective tool for triaging patients in public and crowded places.

## MATERIALS AND METHODS

This study was conducted at the 22nd Bahman Hospital of Qazvin, Iran, psychiatric clinic. The participants of this study included outpatients receiving psychiatric services from 2020 to 2021. Volunteers who underwent clinical evaluation were consecutively enrolled in the study. This study complied with the research priorities of Qazvin University of Medical Sciences and was approved by the institutional ethics committee. Entry criteria encompassed age above 13 years, giving written consent, and lack of severe and acute symptoms. Thus, patients who required emergency care were excluded from the study.

Demographic data (age, sex, education, marital status) and patients' clinical conditions were recorded in particular forms. Other data‐collection tools included the Symptom Checklist‐90‐Revised (SCL‐90‐R) and Kuppuswamy's Socioeconomic Status Scale. Participants were evaluated and followed up by a psychiatry resident based on a standard protocol to identify and prevent adverse clinical outcomes.

The SCL‐90‐R is a popular tool for screening psychopathological symptoms and diagnosing and predicting psychological diseases. This questionnaire contains 90 queries that can be responded to within 12 min and measures primary symptoms, including somatization, obsessive–compulsive disorder (OCD), interpersonal sensitivity, depression, anxiety, hostility, phobic anxiety, paranoid ideation, and psychoticism. The SCL‐90‐R was developed by Derogatis et al.^18^, and its validity has been reported to be 0.77 by Cronbach's *α* coefficient. The reliability of this questionnaire has been reported as 0.8–0.9 using the test–retest method.^19^ In Iran, Anisi et al.^20^ reported Cronbach's *α* coefficient of this questionnaire ranged from 0.75 to 0.92 for its subscales and was reported to be 0.98 for general indicators. For each symptom, the total scores obtained were divided by the number of relevant questions. For general symptoms, the sum of the scores obtained was divided by the total number of questions multiplied by 100. Based on this questionnaire, a score of 90–200 indicates a major problem, and a score above 200 reflects a serious psychological condition.

The patients' socioeconomic status was assessed using Kuppuswamy's Socioeconomic Status Scale, in which education, job, and monthly income are considered.^21^ In this research, we used the Persian version of this questionnaire, which has good reliability (Cronbach's *α* of 0.83).^22^

Based on Question 15 of the SCL‐90‐R, patients were divided into two groups: with suicidal thoughts and without suicidal thoughts. This item questioned if the participants had ideation about terminating their lives. Although this statement may not discriminate between those with or without the intention of ending their lives, it is a good indicator of suicidal ideation at a critical phase when the risk of suicide continues to be a serious concern. After confirming the presence of suicidal ideation, the psychiatry resident performed further clinical evaluations to verify suicidal thoughts in the participants and to ensure their correct grouping. In the next step, demographic, socioeconomic, and clinical data were compared between the two groups.

### Statistical analysis

After collecting the data, they were analyzed by SPSS Version 25. Categorical variables were presented by frequency and percentage, and continuous variables by mean and standard deviation. The independent sample Student's *t*‐test and *χ* ^2^ test were used to compare continuous and categorical variables between the groups, respectively. A binary multiple logistic regression model was developed to assess the predictive values of the demographic, socioeconomic, and clinical factors identified for suicidal ideation. The statistical significance level was considered as *p* value < 0.05.

## RESULTS

Out of 336 participants who completed the questionnaires, only 288 were analyzed, as 48 questionnaires were excluded due to incomplete information. Among the respondents, 78 individuals had suicidal thoughts, while 210 showed no signs of suicidal ideation. Table 1 presents the demographic, socioeconomic, and clinical characteristics of the respondents. Table 1 demonstrates the respondents' demographic, socioeconomic, and clinical features.

**Table 1:** The participants’ demographic, socioeconomic, and clinical characteristics based on the presence or absence of suicidal thoughts.

Variables	With suicidal thoughts		Without suicidal thoughts		*p*	
Age (years)	40.05 ± 14.83		38.13 ± 16.43		0.36	
*Sex*						
Male	36 (46.2%)		72 (34.3%)		0.06	
Female	42 (53.8%)		138 (65.7%)			
*Marital status*						
Single	38 (48.7%)		82 (39.0%)		0.01	
Married	23 (29.5%)		100 (47.6%)			
Divorced	17 (21.8%)		28 (13.3%)			
*Education*						
Below diploma	42 (53.8%)		62 (29.5%)		0.001	
Diploma	19 (24.4%)		79 (37.6%)			
Academic	17 (21.8%)		69 (32.9%)			
*Socioeconomic class*						
Low	56 (71.8%)		98 (46.7%)		0.001	
Middle	12 (15.4%)		73 (34.8%)			
High	10 (12.8%)		39 (18.6%)			
*Clinical diagnosis*						
Personality disorder	18 (23.1%)		28 (13.3%)		0.004	
Major depression	29 (37.2%)		58 (27.6%)			
Mood disorder	16 (20.5%)		43 (20.0%)			
Bipolar disorder	10 (12.8%)		28 (13.3%)			
Other disorders	5 (6.4%)		54 (25.7%)			

*Note*: Data are expressed as *n* (%) and mean ± standard deviation.

Women predominated in both study groups, with and without suicidal thoughts (53.8% and 65.7%, respectively), and the mean ages in these groups were 40.05 ± 14.83 and 38.13 ± 16.43 years, respectively (Table 1; *p* > 0.05). The two groups had statistically significant differences in marital status, education, and socioeconomic status (Table 1; *p* < 0.05). Also, psychiatric diagnostic indicators showed considerable differences between the two groups, so major depression, personality disorders, mood disorders, and bipolar disorder were significantly more prevalent among those with suicidal thoughts (Table 1; *p* < 0.05).

Regarding psychological symptoms, hostility, anxiety, depression, paranoid ideation, phobic anxiety, psychoticism, somatization, interpersonal sensitivity, OCD, and total SCL‐90‐R symptoms were significantly more common in individuals who had suicidal thoughts compared to those without suicidal thoughts (Table 2; *p* < 0.05).

**Table 2:** Comparison of psychopathological factors between respondents based on the presence or absence of suicidal thoughts.

Main symptoms	With suicidal thoughts	Without suicidal thoughts	*p*
Hostility	1.52 ± 0.42	1.03 ± 0.54	<0.001
Depression	2.07 ± 0.47	1.31 ± 0.52	<0.001
Anxiety	2.36 ± 0.47	1.77 ± 0.62	<0.001
Paranoid ideation	1.72 ± 0.56	1.08 ± 0.46	<0.001
Phobic anxiety	1.21 ± 0.61	0.78 ± 0.35	<0.001
Psychoticism	2.07 ± 0.78	0.74 ± 0.44	<0.001
Somatization	1.97 ± 0.39	1.33 ± 0.42	<0.001
Interpersonal sensitivity	1.27 ± 0.39	0.90 ± 0.39	<0.001
Obsession	2.06 ± 0.28	1.16 ± 0.53	<0.001
Total SCL‐90‐R symptoms	150.84 ± 37.87	119.13 ± 33.81	<0.001

*Note*: All values are mean ± standard deviation. All psychiatric symptoms listed were significantly associated with suicidal thoughts (*p* < 0.001).

Abbreviation: SCL‐90‐R, Symptom Checklist‐90‐Revised.

Table 3 shows the correlation between SCL‐90‐R items and suicidal thoughts, indicating that OCD, psychoticism, depression, and somatization had the strongest correlation coefficients with suicidal ideation (*p* < 0.001).

**Table 3:** The correlation of SCL‐90‐R parameters with suicidal thoughts.

Variables	*r*	*p*
Obsessive–compulsive disorder	0.640**	0.000
Anxiety	0.410**	0.000
Psychoticism	0.730**	0.000
Depression	0.549**	0.000
Paranoid ideation	0.498**	0.000
Interpersonal sensitivity	0.384**	0.000
Phobic anxiety	0.401**	0.000
Somatization	0.562**	0.000
Hostility	0.384**	0.000

Abbreviation: SCL‐90‐R, Symptom Checklist‐90‐Revised.

**Indicates p < 0.01, denoting a highly significant correlation.

Table 4 summarizes the association between suicidal ideation and demographic, socioeconomic, and clinical variables. As can be seen, suicidal ideation showed a significant relationship with the SCL‐90‐R score (odds ratio [OR] = 1.04, 95% confidence interval [CI]: 1.02–1.05), being married (OR = 0.13, 95% CI: 0.05–0.29), lower than diploma education (OR = 2.95, 95% CI: 1.17–7.57), and low socioeconomic class (OR = 5.80, 95% CI: 1.68–20.44) (Table 3; *p* < 0.05). In addition, the participants who suffered from a personality disorder (OR = 3.86, 95% CI: 1.03–14.63) and schizophrenia (OR = 6.40, 95% CI: 1.89–22.42) had higher odds of having suicidal ideation compared to those who had other disorders.

**Table 4:** The relationship of suicidal thoughts with demographic, socioeconomic, and clinical variables.

Variables	*B*	OR [95% CI]	*p*
SCL‐90‐R	0.04	1.04 [1.02–1.05]	<0.001
*Marital status (Ref: being single)*			
Married	−2.04	0.13 [0.05–0.29]	<0.001
Divorced	−0.53	0.59 [0.24–1.44]	0.24
*Education (Ref: Academic education)*			
Below diploma	1.09	2.95 [1.17–7.57]	0.02
Diploma	0.63	1.86 [0.65–5.39]	0.23
*Clinical diagnosis (Ref: other disorders)*			
Personality disorder	1.36	3.86 [1.03–14.63]	0.04
Schizophrenia	1.87	6.40 [1.89–22.42]	0.003
Mood disorder	0.27	1.31 [0.34–5.01]	0.68
Bipolar disorder	1.06	2.86 [0.75–11.13]	0.12
*Socioeconomic status (Ref: high class)*			
Low class	1.77	5.80 [1.68–20.44]	0.005
Middle class	0.22	1.25 [0.34–4.51]	0.73
Gender (female)	0.12	1.12 [0.58–2.19]	0.72
Age	0.002	1 [0.98–1.02]	0.86

Abbreviations: CI, confidence interval OR, odds ratio; SCL‐90‐R, Symptom Checklist‐90‐Revised.

## DISCUSSION

This study was an effort to identify the predictors of suicidal thoughts. There are signs of suicide, and being aware of them is necessary not only for specialists but for every member of human society. Identifying individuals having suicidal thoughts is of particular importance, as 9.3% of these subjects commit suicide.^23^ Therefore, we investigated the relationship between suicidal thoughts and demographic, socioeconomic, and psychological health parameters in outpatients receiving psychiatric services. The results showed that suicidal thoughts were significantly associated with a high SCL‐90‐R score, low education, and socioeconomic problems.

Initially, we assessed the impact of demographic variables on suicidal ideation, and the results delineated education level as one of the factors affecting suicidal thoughts. The participants who had below diploma education had 2.95 higher odds of developing suicidal thoughts than those who had academic education. Also, Li et al. reported similar results in their study, asserting that suicide was less frequent in those with academic education.^24^

In the present study, another demographic determinant of suicidal thoughts was marital status. In this regard, suicidal thoughts were 7.69 times more common in single than in married subjects. There was no significant difference between single subjects and those who were separated from their spouses. These results were in line with the results of Mohammadinia et al., who reported that suicidal ideation was more prevalent among single individuals compared to married subjects.^25^ Shafiee‐Kandjani et al. showed that suicidal thoughts were most common among single subjects who had diplomas or lower education.^26^ In the present study, there was no significant difference between subjects who held a university degree and those with a diploma in terms of suicidal ideation. In contrast to the present study, Saiadrezaei et al. showed that suicidal thoughts were more prevalent among married subjects.^27^ This controversy among studies can be related to cultural and socioeconomic circumstances.

Accordingly, we here investigated not only demographic variables but also the socioeconomic status of the participants. Our results showed that a low socioeconomic class increased the risk of nurturing suicidal thoughts by 5.80 times compared to a high socioeconomic class. There is controversy regarding the impacts of socioeconomic factors on suicidal thoughts; however, most studies have reached the conclusion that low income is a risk factor for suicidal behaviors.^28^ Moreover, the results of Rehkopf et al. declared that the rate of suicide was the lowest in societies with high socioeconomic status, reporting that suicide was directly associated with unemployment and inversely with education and occupation, which was in line with the results of the present research.^29^ In this study, low socioeconomic status was identified as a significant predictor of suicidal ideation (OR = 5.80, 95% CI: 1.68–20.44, *p* = 0.005). Specific socioeconomic challenges faced by the study population, such as high unemployment rates, limited access to mental health services, and economic instability exacerbated by inflation and sanctions, may contribute to this finding. For example, many participants with low socioeconomic status reported struggling with job insecurity and the inability to afford basic necessities, which may lead to feelings of hopelessness and despair. Additionally, the lack of affordable housing and educational opportunities further compounds these challenges, particularly for individuals with lower levels of education (OR = 2.95, 95% CI: 1.17–7.57, *p* = 0.02). These results are consistent with previous research in Iran, which has highlighted the impact of economic hardship on mental health outcomes.^30^, ^31^ On the other hand, poor socioeconomic status and low education can sometimes lead to a decrease in life quality and distorted psychological health.^32^, ^33^ Addressing these socioeconomic issues through policy interventions, such as job‐creation programs, affordable mental health care, and social support systems, could play a crucial role in reducing the prevalence of suicidal ideation in this population.

Regarding the diagnostic parameters influencing suicidal thoughts, personality disorder and schizophrenia were identified to be adverse predictors of suicidal ideation. This observation somewhat agrees with the results of Zare et al., who showed that depression, schizophrenia, and antisocial personality disorder had the highest frequencies in subjects with suicidal ideation.^34^ Also, Shafiee‐Kandjani et al. showed that subjects committing suicide suffered from at least one personality disorder and identified depression as the first sign of maladaptive personality disorder among Iranians committing suicide.^26^ Therefore, it can be concluded that psychological disorders are one of the predictors of suicidal thoughts. For instance, patients suffering from schizophrenia, if left untreated, can develop deregulated emotions, leading to the formation of suicidal thoughts.^35^, ^36^

We found that the total SCL‐90‐R score was another predictor of suicidal ideation in the patients referred to our psychiatric clinic. This index, along with other demographic and socioeconomic factors, can be a suitable tool for screening subjects with suicidal thoughts. Among the subscales of the SCL‐90‐R, OCD, Psychoticism, Depression, and Somatization had the highest correlation coefficients with suicidal thoughts.

All psychiatric symptoms examined in this study showed significant correlations with suicidal ideation. However, the strength of these correlations varied among different symptoms. For instance, while symptoms such as psychoticism and depression had the highest correlation coefficients with suicidal thoughts, other symptoms like interpersonal sensitivity, phobic anxiety, and aggression showed comparatively lower correlations. These results emphasize the complexity of suicidal thoughts and the necessity for a multifaceted approach in their assessment.

As the SCL‐90‐R can be completed in 12 min, it can provide a suitable tool for screening subjects in public and crowded places. This issue is particularly important in the triage of patients and can improve the performance of psychiatrists during immediate or delayed patient visits. Learning skills such as problem‐solving and resilience, especially when a person faces crises and feels pain and distress, can improve negative emotion regulation, anger management, and the control of instant behaviors, reducing suicidal thoughts.^35^, ^37^

In line with these results, Chaudhary et al. showed that obsession was associated with a high risk of suicidal thoughts, and depression and hopelessness were also significantly associated with suicidal ideation.^35^ Therefore, in order to avert the risk of suicide, it is necessary to screen subjects suffering from obsessions, depression, psychosis and other psychological disorders in terms of suicidal thoughts. In some cases, the disorders mentioned may not be related to the level of literacy, socioeconomic class, and cultural features, but leaving them untreated may trigger the creation of suicidal thoughts in an individual.

Addressing the limitations of this study, it had a cross‐sectional design and was conducted on a clinical sample recruited by taking into consideration all known precautions. Therefore, more studies are required in this field to be able to generalize the results. Although 288 participants were enrolled in this study, which is an appropriate and proportionate sample size, only 27.1% of these individuals declared that they had suicidal thoughts, limiting the predictive value of the parameters identified. In the future, it is recommended to conduct large‐scale studies on a bigger sample size. The study's location (i.e., a single psychiatric care clinic) also limits the generalization of the results to other places and cultures.

Another limitation of this study is the exclusion of patients with severe and acute psychiatric symptoms, who may be at higher risk for suicidal behavior. Including such patients could potentially lead to increased risk and ethical concerns, as their severe symptoms might require immediate intervention and specialized care, which could confound the study results. This exclusion may limit the generalizability of our results to the broader population of psychiatric patients, particularly those with severe mental disorders. Future studies should aim to include a more diverse sample, including individuals with severe symptoms, to better understand the full spectrum of factors associated with suicidal ideation. While this study focuses on the presence or absence of suicidal ideation at a specific point in time, it is important to consider how these thoughts may evolve and potentially lead to suicidal behavior. Future longitudinal studies are needed to explore the trajectory of suicidal ideation and its transition to actionable behaviors. Clinicians should monitor patients with suicidal ideation over time, as the risk of progression to suicidal behavior may increase with untreated or worsening psychopathology.

Conditions where suicidal ideation is a diagnostic criterion, such as depression, may not be directly comparable to other psychiatric conditions. This could introduce bias in the analysis, and future studies should consider stratifying analyses based on diagnostic categories to account for these differences. Clinicians should also be aware of these diagnostic nuances when interpreting the results of studies on suicidal ideation.

The validity of psychiatric diagnoses in this study may be limited, particularly in high‐risk suicide patients, where clinical diagnoses are often challenging to establish. Future studies should consider incorporating more comprehensive diagnostic tools or longitudinal assessments to improve diagnostic accuracy. Additionally, the use of structured clinical interviews or multidisciplinary diagnostic evaluations could enhance the reliability of psychiatric diagnoses in this population.

The results of this study highlight the significant role of psychopathological symptoms, socioeconomic status, and demographic factors in suicidal ideation among Iranian outpatients. However, it is important to consider how these factors may differ or align across various cultural and linguistic contexts. For instance, studies in Western cultures, such as the United States and Europe, have similarly identified depression, low socioeconomic status, and marital status as key predictors of suicidal ideation.^38^, ^39^ In contrast, studies in East Asian cultures, such as Japan and South Korea, have emphasized the role of collectivist societal pressures and academic stress as additional risk factors.^40^, ^41^ These cultural differences suggest that while certain risk factors for suicidal ideation may be universal, their relative importance and expression can vary significantly across societies. Future cross‐cultural studies are needed to further explore these variations and develop culturally sensitive interventions.

## CONCLUSIONS

Our results showed that suicidal thoughts pose a serious threat in the outpatients referred to psychiatric clinics and are associated with SCL‐90‐R subscales, especially OCD, Psychoticism, Depression, and Somatization. In addition, personality disorder, schizophrenia, poor socioeconomic status, low education, and being single were identified to be predictors of suicidal thoughts. It is recommended to bring screening for suicidal thoughts under attention in psychiatric clinics and implement appropriate measures to prevent suicide. The timely treatment of psychiatric disorders, planning to improve the economic condition, and implementing training programs can play a key role in preventing suicide. Based on the study results, we recommend close monitoring of psychiatric disorders, emotional/behavioral problems, and academic performance declines. On the other hand, nongovernmental organizations, benefactors, and authorities should implement measures to empower families economically and financially to reduce suicidal thoughts in society.

## AUTHOR CONTRIBUTIONS

**Alireza Haji Seyed Javadi:** Methodology; visualization; conceptualization; investigation; software; data curation; writing—original draft. **Adeleh Askari Diarjani:** Conceptualization; writing—reviewing and editing. **Ali Akbar Shafikhani:** Conceptualization; methodology; software; visualization; investigation; supervision; writing—reviewing and editing.

## CONFLICT OF INTEREST STATEMENT

The authors declare no conflicts of interest.

## ETHICS APPROVAL STATEMENT

This study was approved by the Ethics Committee of Qazvin University of Medical Sciences.

## PATIENT CONSENT STATEMENT

Written informed consent was obtained from the patients for publication of this information.

## CLINICAL TRIAL REGISTRATION

N/A.

## Data Availability

All relevant data are within the paper.
